# A National Board of Health

**Published:** 1895-03

**Authors:** 


### A NATIONAL BOARD OF HEALTH.

Medical men and the medical press have for a long time been urging the establishment of a National Board of Health as a means of securing public protection against epidemic disease. The suggestion to make this department of our public hygiene responsible to a Medical Secretary in the President's Cabinet does not seem to have met with favor in Washington.

The President, however, recognizes the necessity for something of this sort, and thus expressed himself in his last regular message: "I am entirely convinced that we ought not to be longer without a National Board of Health or national health officer charged with no other duties than such as pertain to the protection of our country from the invasion of pestilence and disease. This would involve the establishment, by such board or officer, of proper quarantine precautions, or the necessary aid and counsel to local authorities on the subject, prompt advice and assistance to local boards of health or health officers in the suppression of contagious diseases, and in cases where there are no such local boards or officers, the immediate direction by the National Board or officer of measures of suppression, constant and authentic information concerning the health of foreign countries and all parts of our own country as related to contagious diseases; and consideration of regulations to be enforced in foreign ports to prevent the introduction of contagion into our cities, and the measures which should be adopted to secure their enforcement. There seems to be at this time a decided inclination to discuss measures of protection against contagious diseases in international conference, with a view of adopting means of mutual assistance. The creation of such a national health establishment would greatly aid our standing in such conferences, and improve our opportunities to avail ourselves of their benefits. I earnestly recommend the inauguration of a National Board of Health or similar national instrumentality, believing the same to be a needed precaution against contagious disease and in the interest of the safety and health of our people."

